# Banter within the NHS: A tool for boosting morale or a front for workplace bullying?

**DOI:** 10.1016/j.fhj.2024.100143

**Published:** 2024-05-11

**Authors:** Lucy Dicks-Ilori, Marianne Morgan, Mengshi Yuan, Sarbjit Clare

**Affiliations:** Sandwell and West Birmingham NHS Trust, Birmingham City Hospital, Dudley Road, Birmingham, West Midlands B18 7QH, UK

## Abstract

Workplace humour, such as banter between colleagues, is a widespread means of developing relationships and relieving daily work stresses. Despite this, banter in the workplace is a prevalent theme of harassment and bullying claims. With staff morale at an all-time low among NHS employees, efforts must be made to identify and rectify issues which work to damage staff experiences within the organisation. We aimed to explore both the positive and negative impacts of banter on NHS staff well-being. We discuss the role of staff training on the appropriate use of workplace humour, with reference to a workshop delivered to NHS employees, educating them on the appropriate use of banter.

## Introduction

Within the NHS, morale has been recently reported as ‘rock-bottom’ among junior trainees,^1^ with a high rate of stress-related sickness in the establishment.^2^ Professional resilience to cope with challenging working environments is often explored in healthcare professionals, with humour being identified as a characteristic that can contribute to adapting to adversity.^3^ Alongside humour being a coping strategy to relieve the stresses of the healthcare environment, it can also promote camaraderie between working colleagues.^4^

A prevalent form of humour utilised in day-to-day human social interaction is banter, defined as ‘teasing or joking talk that is amusing and friendly’.^5^ Thought to have originated from London street slang in the 17th century, initially meaning ‘good-humoured ridicule’,^6^ its modern-day use focuses on conversation that is ‘light-hearted’ and ‘witty’.^7^ Banter is a widespread means of humoured communication between colleagues, with 98% of 1,025 participants surveyed by the Institute of Leadership and Management in 2018 responding that they had experienced banter in the workplace.^8^ Banter can improve teamwork and cohesiveness, offering respite in monotonous work days and allowing an outlet in stressful environments.^9^ Furthermore, humour exchanged between different levels of workers may aid in diffusing the barriers often engrained in hierarchies within work companies.^9^

Nevertheless, teasing remarks, often delivered without ill intent, can be delivered with negative connotations, leading to potential workplace bullying.^10^ ‘It was just a bit of banter' is a phrase often used to justify harmful and humiliating behaviour, while 'banter' being used as a defensive excuse in employment tribunals is on the rise.^11^ As per the Institute of Leadership and Management survey, 20% reported having been embarrassed by workplace banter, 10% avoided circumstances at work after being the subject of banter, and 4% quit their jobs as a result.^8^

Recognising and addressing the negative impacts of inappropriate banter is of significance within the NHS, where bullying remains a prevalent issue. A 2021 NHS staff survey showed that in the last 12 months, 11.6% and 18.7% of respondents had experienced ‘harassment, bullying or abuse’ from their managers or other colleagues, respectively.^12^ Behaviours such as harassment and bullying in the workplace have been shown to increase rates of poor mental health, job dissatisfaction, sickness absences and thoughts about quitting amongst victims.^13^ This, in turn, has organisational implications for workforce productivity and retention. In a climate where the NHS faces dwindling resources, strategies targeting the problem of workplace bullying should be a particular area of focus.

Research addressing banter, specifically within the NHS, is sparse. The importance of ensuring safe and enjoyable working environments to positively impact healthcare professionals’ well-being and ability to deliver optimum care to patients cannot be underestimated. Therefore, the Women's Clinician Network at Sandwell and West Birmingham NHS Trust ran a workshop addressing whether banter should be removed within the NHS, alongside introducing a checklist to ensure that workplace banter within the NHS is appropriate and has positive outcomes on the working environment.

## Methods

An interactive workshop was delivered to a group of healthcare workers and held at Sandwell and West Birmingham NHS Trust. This national event, titled ‘Stand with me, not by me. Be an ally, don't bystand but upstand,’ was open to all NHS employees free of charge (Appendix A). A total of 45 delegates from across the UK attended, with 80% of the audience consisting of consultants and doctors in training. The remaining 20% of attendees included nurses, hospital management personnel and allied health professionals. Five of the 45 attendees were male. Participants were surveyed on whether banter should be banned within the NHS before and after completing the workshop to assess NHS workers’ attitudes towards banter within the working environment. Consent was required from all participants.

### *Workshop details*

During the session, participants were educated on workplace banter, including its role in boosting morale. The harmful use of banter was also covered. Emphasis was placed on recognising protected characteristics covered in the Equality Act (2010), section 26, that should not be the subject of banter.

A Banter Safety Checklist was devised to ensure the appropriate use of banter in the workplace (Box 1).

Box 1. The ‘Banter Safety Checklist’ taught to participants of ‘Stand with me, not by me. Be an ally, don't bystand but upstand’ workshop.

**Banter Safety Checklist**

For banter to be 'safe' one needs to ensure the following three criteria are met:

1. **Everyone** involved understands it is banter

2. **Everyone** finds it funny

3. **Everyone** there feels included and safe, ie when close friends or colleagues joke or tease each other, and all enjoy it

Alt-text: Unlabelled box

Fictional case studies were created for participants to practise and discuss using the Banter Safety Checklist to differentiate safe and inappropriate behaviour. We include two case examples utilised in the workshop to demonstrate how to differentiate banter from bullying using the ‘Banter Checklist’.

Box 2. Fictional case study 1 delivered to participants of ‘Stand with me, not by me. Be an ally, don't bystand but upstand’ workshop

**Case 1**

*A junior doctor, Sandra, has a very stressful day looking after sick patients on the ward and running an overbooked clinic. She confides in her supervisor that she feels overwhelmed and during the discussion she becomes emotional and sheds some tears.*

*Following on from this the supervisor makes repeated remarks in front of the team such as ‘He can't ask Sandra to see another referral otherwise she might cry again…’*

*She asks her supervisor to stop making these comments; however, other members of the team responded by telling her ‘it's a bit of banter and you need to lighten up’*

Alt-text: Unlabelled box

Using the banter checklist, case 1 demonstrates an inappropriate use of banter (Box 2). Under immense pressure from the overwhelming workload, Sandra confided in her supervisor to seek support and guidance, only to be ridiculed in front of the team. Banter is only safe when everyone finds it funny; however, in this case, Sandra feels humiliated and hurt by her supervisor's remark. Instead of feeling included and part of the team, Sandra feels isolated. This would have a detrimental effect on the team dynamic and the rapport between Sandra and her supervisor.

Box 3. Fictional case study 2 delivered to participants of ‘Stand with me, not by me. Be an ally, don't bystand but upstand’ workshop

**Case 2**

*Malik is a specialist registrar in geriatric medicine. Having been to the coroner's court in the past due to a junior documenting incorrectly in a patient's notes, he always documents his own ward round entries.*

*He understands that this adds to the ward round time but often shares his experience and advocates that his junior doctors pay attention to their documentation. The ward round can often take 3–4 h when Malik leads it as he is very thorough with his work.*

*On Monday morning, he is leading the ward round and the team manages to finish in 2.5 h (which is record time for Malik). Malik jokes that the team must go for a team coffee to celebrate his rare achievement.*

Alt-text: Unlabelled box

Case 2 is a good demonstration of safe banter (Box 3). The whole team finds it funny, and no one feels excluded or hurt by Malik's remark about finishing the ward round early. All three criteria from the ‘Banter Safety Checklist’ are met.

## Results

**Fig. 1:**
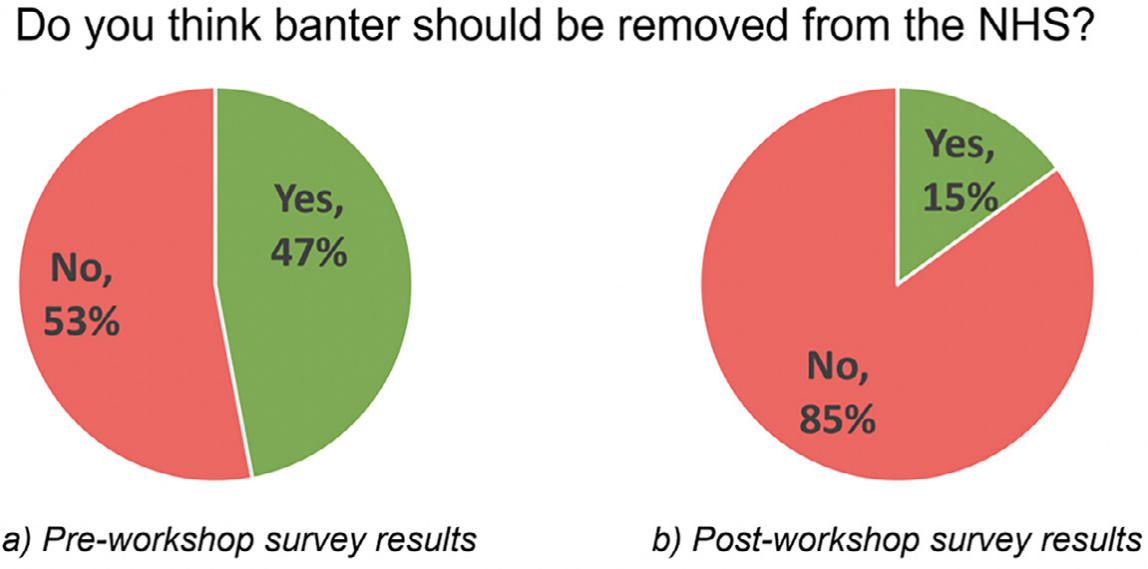
Pie charts demonstrating survey respondent answers to whether banter should be removed from the NHS pre and post workshop completion.

Of the 45 attendees, 15 and 13 delegates responded to the pre- and post-workshop surveys, respectively. Prior to the course, there were mixed views on whether banter should be banned within the NHS, with 53% (*n* = 8) voting no and 47% (*n* = 7) voting yes. Post-course, the proportion of respondents voting to keep banter within the NHS had increased to 85% (*n* = 11) (Fig. 1).

## Discussion

We aimed to assess the perceptions around banter and its place within the NHS among a selection of healthcare workers. A workshop was then delivered, educating participants on how banter can be used safely and in a way that protects the dignity of others.

Our initial survey conducted at the start of the workshop demonstrates the varied experiences and perceptions of banter among professionals. However, following the introduction of the ‘Banter Safety Checklist’, there was a greater increase in participants wanting to keep banter in the NHS, perhaps demonstrating that banter can positively impact working environments if used correctly and safely.

Banter as a positive communication tool between colleagues is evident in the literature. As determined in a meta-analysis by Mesmer‐Magnus J *et al*. (2012), positive humour in the workplace can improve both the physical and mental health of employees, boost productivity and aid in counteracting workplace stress.^14^ Positive perceptions of banter as a means of workplace humour have been echoed in other workplaces, such as the I.T. industry, allowing employees to foster social networks and aiding in the development of an enjoyable culture within the organisation.^15^ 73% of Institute of Leadership and Management members reported they would not ban workplace banter, with respondents finding banter a medium to develop an enjoyable environment, fostering positive engagement with work colleagues.^8^ Nevertheless, inappropriate banter can negatively impact several personal aspects, including mental health and confidence, alongside affecting personal performance at work.^16^

A key component of the workshop was introducing participants to the ‘Banter Safety Checklist’. This tool was devised with the aim of assisting participants in differentiating appropriate and inappropriate banter. The components of the checklist draw on factors that may lead to banter being perceived negatively or as harassment. The Equality Act 2010, Section 26, defines harassment as 'any unwanted conduct related to a relevant protected characteristic', or of a sexual nature, 'and which violates a person's dignity or has the purpose or effect of creating an intimidating, hostile, degrading, humiliating or offensive environment'.^17^ Therefore, for everyone to feel safe and to find banter funny, participants should be mindful that their jokes do not infringe on the dignity and respect of others. Inclusivity and reciprocity were also features of the banter safety checklist. Studies suggest that when participants feel excluded or do not know each other well, people are more likely to interpret 'banter' as a negative experience or as bullying.^15^^,^^18^ 'Knowing your audience' and approaching social interactions with an inclusive attitude can help to prevent situations where banter is experienced negatively. The resulting increase of those voting to keep banter within the NHS after our course indicates that providing people with a framework in which banter can be used safely can increase the perception of its use as a positive tool to improve morale, support colleagues and demonstrate care for them.

Increasing awareness through education about banter can improve the ability to recognise and challenge inappropriate behaviour within the NHS. Instances of harassment and bullying are generally underreported in the NHS, with only 48.7% of targeted individuals or bystanders stating that they had done so after experiencing this behaviour.^12^ A qualitative study on workplace bullying in the NHS found that teasing and sarcasm were significantly less likely to be reported (3.2%) than other, more overt forms of bullying, such as physical abuse (14.3%).^19^ This could indicate a lack of understanding among NHS employees around what constitutes workplace bullying or a willingness to 'let things slide' where examples of banter have the potential to be perceived as less harmful. Based on our findings, clear guidance and structured training within the NHS should be used to educate employees about the negative impact their words and actions can have on others, with emphasis placed on the appropriate use of banter.

Our workshop, as a means of educating NHS staff about the appropriate use of banter, was received positively by attendees. The ‘Chatham House Rule’ was employed throughout the session, allowing participants to share vulnerable experiences and confide openly with each other about sensitive issues of harassment and bullying at work. Speakers included hospital management, which had the advantage of bridging the gap between organisation and employee.

The conclusions that can be drawn from our data have potential limitations. Our sample size was small at 45, and the engagement rate amongst attendees with the pre- and post-course surveys was low. While this sample size was adequate for a pilot study and snapshot view of current perceptions around banter, future educational initiatives should be delivered on a larger scale to improve the generalisability of results. Additionally, the audience was predominantly female and, therefore, needed to be more representative of the NHS workforce. Future workshops could highlight any differing views on workplace banter between sexes. A further limitation was the voluntary nature of our workshop, which lent itself to self-selection bias, where participants were more likely to be interested in the subject matter and to engage positively with the course content. Mandatory training required of NHS employees could be expanded to include workplace banter to ensure a wider audience is reached.

Overall, promoting a culture of openness and respect in the workplace is key to protecting the well-being of employees. Methods of relieving stress and building meaningful relationships between workers are vital in a demanding environment such as the NHS, whilst recognising and addressing the negative impacts of banter on the workplace is essential to promote an inclusive and respectful environment for its workers. Using humour through banter can have a positive impact; conversely, it can be immensely damaging when misused. Work must be done to raise awareness of this issue to initiate a behavioural shift amongst NHS employees. Leaders within the organisation should ensure that healthcare workers are appropriately trained in workplace etiquette, including recognising and escalating undermining behaviour. We advocate for staff training to reference banter specifically and for the ‘banter checklist’ to be used and shared by all colleagues.

## Declaration of competing interest

The authors declare that they have no known competing financial interests or personal relationships that could have appeared to influence the work reported in this paper.

## Appendix Supplementary materials

Image, application 1
