# Supplementary material for: Banter within the NHS: A tool for boosting morale or a front for workplace bullying?
Source: Future Healthc J. 2024 May 11;11(2):100143. doi: 10.1016/j.fhj.2024.100143 (PMC11166689; doi:10.1016/j.fhj.2024.100143)
Supplement: Supplementary file 1 [file mmc1.docx]

**Appendices**

Appendix A:
